# Prophylactic administration of tranexamic acid combined with thromboelastography-guided hemostatic algorithm reduces allogeneic transfusion requirements during pediatric resective epilepsy surgery: A randomized controlled trial

**DOI:** 10.3389/fphar.2022.916017

**Published:** 2022-08-17

**Authors:** Ting Zhang, Hua Feng, Wei Xiao, Jingsheng Li, Qinghai Liu, Xuexin Feng, Dezhou Qi, Xiaotong Fan, Yongzhi Shan, Tao Yu, Guoguang Zhao, Tianlong Wang

**Affiliations:** ^1^ Department of Anesthesiology, Xuanwu Hospital, Capital Medical University, Beijing, China; ^2^ Department of Neurosurgery, Xuanwu Hospital, Capital Medical University, Beijing, China; ^3^ Department of Functional Neurosurgery, Xuanwu Hospital, Capital Medical University, Beijing, China

**Keywords:** tranexamic acid, antifibrinolytics, thromboelastography, epilepsy surgery, pediatric anesthesia, transfusion, blood loss, coagulation therapy

## Abstract

**Background:** Intraoperative bleeding and allogeneic transfusion remain common problems in pediatric resective epilepsy surgery. Tranexamic acid (TXA) is a widely recommended antifibrinolytic drug that reduces blood loss and transfusion requirements for bleeding patients. Thromboelastography (TEG)-guided hemostatic algorithm is commonly used in bleeding management. This trial was designed to validate the efficacy of a multimodal coagulation therapy involving continuous TXA infusion with TEG-guided hemostatic algorithm in reducing allogeneic exposure risk in pediatric resective epilepsy surgery.

**Methods:** Eighty-three children undergoing resective epilepsy surgery were randomized into a treatment group (Group T; *n* = 42) and a control group (Group C; *n* = 41). Group T received prophylactic TXA (10 mg/kg followed by 5 mg/kg/h) with TEG-guided hemostatic algorithm, whereas Group C received conventional coagulation management. The primary outcome was allogeneic transfusion rate during surgery, and the secondary outcomes were intraoperative blood loss, incidence of postoperative seizures, and thromboembolic events during hospitalization.

**Results:** The incidence of intraoperative allogeneic transfusion reduced by 34.7% with the use of a multimodal coagulation therapy (19.0% in Group T vs. 53.7% in Group C; RR 0.355, 95% CI 0.179–0.704; *p* = 0.001). This was mainly triggered by a significant reduction (44.1%) in intraoperative plasma transfusion (7.1% in Group T vs. 51.2% in Group C; RR 0.139, 95% CI 0.045–0.432; *p* = 0.000). The risk of intraoperative RBC transfusion was lower in Group T than in Group C, but the difference was not statistically significant (14.3% in Group T vs. 29.3% in Group C; RR 0.488, 95% CI 0.202–1.177; *p* = 0.098). No platelets were transfused in both groups. Further, 19 (45.2%) patients in Group T received fibrinogen concentrates guided by TEG data, whereas 1 (2.4%) patient in Group C received fibrinogen concentrates empirically. There were no significant differences in estimated blood loss and postoperative seizures between the two groups, and no thromboembolic events were observed after surgery.

**Conclusion:** Prophylactic administration of TXA combined with TEG-guided hemostatic algorithm can be an effective multimodal coagulation strategy for reducing allogeneic transfusion requirements during pediatric resective epilepsy surgery.

**Clinical Trial Registration:** www.chictr.org.cn/index.aspx, identifier ChiCTR1800016188.

## Introduction

Studies have shown that surgical treatment is valuable for certain children with medically intractable epilepsy (Sperling et al., 2016; Dwivedi et al., 2017). However, considerable blood loss and coagulation disorders are important risk factors that can compromise the safety of pediatric resective epilepsy surgery (Thudium et al., 2014). Children are associated with low absolute blood volume and relatively larger head-to-body ratio (Livingston and Lee, 2000), which made pediatric intracranial surgeries at high risk of intraoperative bleeding. Moreover, preoperative exposure to antiepileptic drugs (AEDs) could cause coagulation disorders, including thrombocytopenia, platelet dysfunction, hypofibrinogenemia, or acquired von Willebrand disease (Finsterer et al., 2001; Gerstner et al., 2006; Pan et al., 2007). In addition, extensive tissue injury during surgery can produce large amounts of tissue activators that activate plasmin from plasminogen, leading to hyperfibrinolysis. The acquired coagulopathy can aggravate intraoperative bleeding and increase allogeneic transfusion requirements (Goobie and Haas, 2014). Therefore, significant efforts should be made to optimize coagulation function and reduce the exposure to allogeneic products during pediatric resective epilepsy surgery.

No standard strategies have been previously recommended to optimize hemostatic systems for pediatric epilepsy surgery, and clinical transfusion practice is primarily based on a combination of subjective bleeding assessment and standard laboratory tests. Given that timely laboratory values cannot be obtained during surgery, allogeneic blood products, especially plasma and platelets, have usually been transfused at the discretion of the attending surgeon and anesthesiologist when significant blood loss was expected or occurred. This empirical transfusion strategy may lead to allogeneic blood abuse and waste and place patients at high risk for transfusion-related adverse outcomes (Stainsby et al., 2008; Lavoie, 2011).

Tranexamic acid (TXA) is an antifibrinolytic drug that competitively inhibits the conversion of plasminogen to plasmin, thereby blocking the proteolytic action of plasmin on fibrin clot, and as such inhibiting fibrinolysis during surgery (McCormack, 2012). Strong evidence has shown that prophylactic TXA use can reduce blood loss and transfusion requirements in surgeries for pediatric scoliosis, cardiac, and craniosynostosis without apparent morbidity or mortality (Dadure et al., 2011; Goobie et al., 2011; Pasquali et al., 2012; McNicol et al., 2016; Goobie et al., 2018). However, few studies have explored the role of TXA in pediatric resective epilepsy surgery.

Thromboelastography (TEG) is a point-of-care test that can be conducted in a relatively short period (15–30 min) and provides a comprehensive, graphical, and numerical evaluation of the patient’s clotting system. TEG can be used to guide transfusion practice during coagulation management, thus reducing the requirement of blood transfusion and improving surgical outcomes (Wikkelso et al., 2016). There is growing evidence supporting the use of TEG to manage surgical bleeding (Wikkelso et al., 2016; Feng et al., 2019; Spahn et al., 2019). However, such evidence has been primarily based on elective cardiac surgery, with limited studies investigating TEG-guided transfusion in craniotomies, especially for children.

In our center, we use a combination of continuous TXA infusion with TEG-guided coagulation management during hemispherectomy; this seems to have reduced the amount of blood loss and subsequent allogeneic transfusion requirements in our cases without promoting clinical complications involving thrombotic events and seizures (Xiao et al., 2016). Therefore, this clinical trial was conducted to assess the efficacy of the multimodal coagulation therapy during pediatric epilepsy surgery. We hypothesized that prophylactic administration of TXA combined with TEG-guided hemostatic algorithm would reduce allogeneic transfusion requirements during pediatric resective epilepsy surgery.

## Materials and methods

### Research design

This single-center, prospective, randomized, controlled clinical trial was conducted at Xuanwu Hospital of Capital Medical University (Beijing, China) from June 2018 to November 2021. The research was approved by the Ethics Committee of Xuanwu Hospital of Capital Medical University [LYS(2018)103] and registered at the Chinese Clinical Trial Registry (www.chictr.org.cn/index.aspx; identifier ChiCTR1800016188). All experimental procedures were conducted in accordance with the Declaration of Helsinki. Written informed consent was obtained from the patient’s parents or legally authorized representatives before enrollment into the study.

### Participants

Patients meeting the following inclusion criteria were included: 1) diagnosis of symptomatic epilepsy, 2) scheduled for elective resection of epileptic focus, 3) aged 1–12 years, 4) body weight ≤40 kg, 5) American Society of Anesthesiologists physical status classification of I or II, and 6) provided written informed consent. The exclusion criteria were as follows: 1) accompanied with liver diseases, 2) accompanied with kidney diseases, 3) accompanied with hematological diseases, 4) family history of hemorrhagic diseases, 5) history of craniotomy, and 6) allergies to tranexamic acid. Patients with a duration of surgery of <3 or >8 h were excluded from analysis given that surgical procedures and consecutive blood loss might differ considerably.

### Randomization and blinding

Enrolled patients were randomized into the treatment group (Group T) or control group (Group C) in a 1:1 ratio using a random digit table provided by a biostatistician at our center *via* SAS software (version 8.02; SAS Institute Inc., Cary, NC, United States). Allocation concealment was ensured by prepackaging the grouping information from the random digit table in sequentially numbered sealed opaque envelopes. The envelopes were kept by a nurse from the postanesthesia care unit who was blinded to this study. When a patient satisfied the inclusion criteria, the nurse opened the corresponding envelope and assigned the patient to the corresponding group. The patients, neurosurgeons, and the outcome assessor (a trained investigator, who collected postoperative data from the Electronic Medical Record System of our center), were all blinded to the group assignment.

### Surgery and anesthesia

All surgical procedures were performed by one of two functional neurosurgeons who had >20 years of experience at our center. Anesthesia management in Group T was performed by a specific anesthesiologist who had been trained to conduct hemostatic strategies according to the protocol. The anesthesiologists who cared for the patients in Group C were assigned randomly according to the arrangement at our department.

All patients received general anesthesia as routinely practiced at our center. The following parameters were consistently monitored: electrocardiography, pulse oximetry, invasive blood pressure (IBP), bispectral index (BIS), nasopharyngeal temperature, partial pressure of end-tidal CO_2_ (P_ET_CO_2_), and urine output. Patients were induced via intravenous propofol (2–3 mg/kg), sufentanil (0.3–0.5 μg/kg), and rocuronium (0.6–1 mg/kg). Thereafter, the patients were intubated and ventilated while maintaining the P_ET_CO_2_ at 30–35 mmHg. When venous access was difficult in the conscious state, intramuscular ketamine (5–7 mg/kg) or sevoflurane administration through inhalation was conducted before induction. Arterial cannulation and central venous puncture were conducted after anesthesia induction. Total intravenous anesthesia was achieved via continuous infusion of propofol (6–10 mg/kg/h) and remifentanil (0.2–0.4 μg/kg/min), which was titrated to maintain a BIS value within 40–60, as well as heart rate and IBP within 20% of baseline. A crystalloid-based fluid was administered to compensate for the preoperative fasting period, basic physiological requirement and intraoperative blood loss, and to maintain hemodynamic stability and urine output of ≥1 ml/kg/h. Moreover, intraoperative heating devices were utilized as necessary to maintain a nasopharyngeal temperature of ≥36°C. Similarly, pH values and plasma calcium levels were optimized to their normal ranges during surgery. Blood salvage was used intraoperatively to collect autologous blood from the surgical site, immediately returning the recovered red blood cells to the patients in real time. To relieve postoperative pain, sufentanil (0.1–0.15 μg/kg) was administered intravenously 30 min before the end of the surgery, and local infiltration with 0.25% ropivacaine (≤1 ml/kg) was performed prior to scalp closure. All patients were extubated at the end of surgery.

Blood-gas analysis (ABL800, Radiometer Medical Aps, Bronshoj, Denmark) was performed at four time points [prior to incision (T1), during the cutting of the dura mater (T2), during suturing of the dura mater (T3), and after scalp closure (T4)], or more frequently as indicated. Red blood cells (RBC) were administered at a target level of 10 g/dl when hemoglobin concentrations obtained from the blood-gas analysis dropped to <8 g/dl. The amount of RBC administered was calculated as follows: amount of RBC (ml) = body weight (kg) × desired increment in hemoglobin concentration (g/dl) × 5 (Morley, 2009).

### Hemostatic strategies in group T

After induction of anesthesia before the surgical incision, an initial bolus of TXA (10 mg/kg) (Changchun Tiancheng Company, Changchun, China) was infused intravenously in 15 min, followed by a continuous infusion at 5 mg/kg/h until the end of the scalp closure. If the cumulative dose of TXA reached 1 g before the end of surgery, the infusion would be stopped.

We employed kaolin-activated TEG analysis (TEG-5000, Haemoscope Corporation, Illinois, United States) to monitor the patient’s coagulation status and guide coagulation component supplementation. All the analyses were performed by an adequately trained anesthesiologist according to standard operating procedures. Whole blood samples for TEG analysis were obtained through a port located at the hub of the arterial catheter near the entry site. Dead space blood, up to 10 ml, was removed to avoid heparin contamination by the arterial flush solution. The collected samples were drawn into 1.8 ml vacutainers containing buffered sodium citrate (0.109 M, 3.2%). At 15 min after obtaining the samples, 1 ml of citrated whole blood was placed into a vial with a kaolin activator (#6300, Haemonetics Corporation) and mixed thoroughly by inversion 5 times. Then, 340 μl of citrated kaolin-activated blood was transferred to a 37°C warmed clear cup containing 20 μl of 0.2 M CaCl_2_ for analysis. The TEG analyzer was allowed to run until LY30 (lysis at 30 min; percentage reduction in the maximal amplitude of the TEG tracing after 30 min) could be determined. The specific TEG parameters that were measured included reaction time (R), coagulation time (K), α angle, maximal amplitude (MA), and LY30.

**FIGURE 1:**
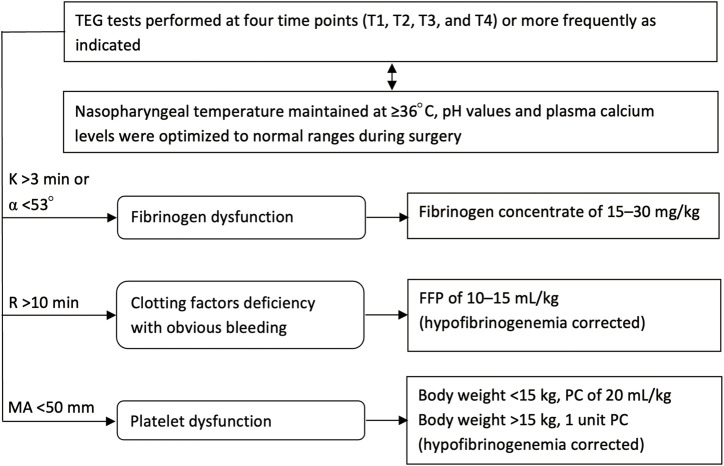
Flow chart for the TEG-guided hemostatic algorithm. TEG, thromboelastography; T1, prior to incision; T2, cutting of the dura mater; T3, suturing of the dura mater; T4, after scalp closure; K, coagulation time; α angle, angle of divergence; R, reaction time to clot formation; MA, maximum amplitude of clot strength; FFP, fresh frozen plasma; PC, platelet apheresis concentrate.

TEG tests were conducted at four time points (similar to that for blood-gas analysis), and additional tests could be performed at any time if necessary, such as if ongoing bleeding had occurred. The TEG-guided hemostatic algorithm is presented in Figure 1. Fibrinogen concentrate (Shanghai RAAS Blood Products Co., Ltd., Shanghai, China) was administered at 15–30 mg/kg when signs of fibrinogen dysfunction (K time >3 min or α angel <53°) were observed. Repeated fibrinogen concentrate was administrated if necessary. After hypofibrinogenemia had been corrected, fresh frozen plasma (FFP) of 10–15 ml/kg was transfused if R time was >10 min with obvious bleeding at the surgical sites, and platelet apheresis concentrate (PC) was administered when MA value was <50 mm. The dose of PC was 20 ml/kg if body weight was <15 kg, otherwise, 1 unit. Generally, supplementation of coagulation components was conducted only if relevant bleeding was observed, but not due to abnormal TEG results without signs of bleeding.

### Conventional hemostatic strategies in group C

Neither TXA nor TEG tests were performed. Hemostatic strategies were selected in accordance with routine practice at our center. Coagulation components were transfused guided by standard coagulation tests, which could be performed at any time as indicated. When prothrombin time (PT) or activated partial thromboplastin time (APTT) prolonged >1.5 times normal, or plasma fibrinogen concentration ranged 1.5–2.0 g/L, FFP of 10–15 ml/kg was administered. Fibrinogen concentrate of 15–30 mg/kg was infused if plasma fibrinogen level was <1.5 g/L. Platelets were transfused if the platelet count dropped to <100 × 10^9^/L. If laboratory coagulation values could not be promptly obtained, hemostatic products were transfused empirically in selected patients mainly depending on signs of microvascular bleeding as well as the patient’s hemodynamic parameters and consultant anesthesiologist’s experience.

### Postoperative management

Postoperative bleeding management was performed at the discretion of the consultant neurosurgical team. Generally, transfusion strategies were selected according to signs of clinical anemia or bleeding and abnormal laboratory values. After surgery, the neurosurgeons made ward rounds twice a day, and examined the patients for symptoms and signs of complications, until the patients were discharged. Side effects, including thrombotic events (e.g., deep vein thrombosis or pulmonary embolism) and seizures, were noted in the Electronic Medical Record System.

### Data collection

Demographic and baseline laboratory data, including age, height, weight, hemoglobin concentration, platelet count, PT, APTT, international normalized ratio (INR), and plasma fibrinogen level, were preoperatively obtained. Surgical data from both groups, such as type of resection, duration of surgery and anesthesia, infusion volume and urine output as well as TEG parameters (R, K, α angle, MA, and LY30) in Group T and standard coagulation parameters in Group C were collected during surgery. Intraoperative blood loss was estimated using standard clinical variables (blood in cell-savers and suction containers and sponge weight). Hemoglobin concentration and platelet count on the first day after surgery, blood products (including RBC, plasma, and platelets) transfused perioperatively, fibrinogen concentrate supplementation, length of hospital stay after surgery, and postoperative complications (including thromboembolic events and seizures) were recorded.

### Outcome measures

The primary outcome was allogeneic transfusion rate during surgery, defined as transfusion of any allogeneic blood product, including RBC, plasma, or platelets. The secondary outcomes included intraoperative blood loss, incidence of postoperative seizures, and postoperative thromboembolic events during hospitalization.

### Sample size calculation

Sample size was calculated according to the incidence of allogeneic transfusion during surgery. In our pilot study (*n* = 32), the allogeneic transfusion rates were 37.50% and 68.75% in Groups T and C, respectively. The sample size software PASS 11 (NCSS, Caseville, Utah, United States) estimated that a total of 39 cases was required for each group to detect a significant change in the allogeneic transfusion incidence, with a type I error *α* = 0.05 and type II error *β* = 0.2. Considering a withdrawal rate of 10%, the target sample size for each group was 43.

### Statistical analyses

The groups T and C were compared with respect to demographics, baseline characteristics, transfusion requirements, and intraoperative and postoperative outcomes. All continuous variables were tested for normality using the Shapiro–Wilk test. Normally distributed data are presented as mean ± standard deviation (SD), and non-normally distributed data as median [25%–75% interquartile range (IQR)]. Continuous data with a normal distribution, such as age, height, preoperative platelet count, PT, INR, duration of surgery and anesthesia, postoperative hemoglobin level, and delta hemoglobin concentration (preoperative to postoperative day 1), were compared between groups by the independent-samples *t*-test. Intragroup comparisons of hemoglobin concentration between baseline and follow-up were analyzed using paired-samples *t*-test. Continuous data not conforming to a normal distribution, including weight, preoperative APTT, infusion volume, urine output, estimated blood loss, postoperative platelet count, and length of hospital stay after surgery, were compared between groups with the Mann–Whitney *U*-test. The Wilcoxon signed-rank test was applied to compare TEG values at different time points in Group T. Categorical variables, such as preoperative anemia (hemoglobin concentration <12 g/dl), preoperative hypofibrinogenemia (plasma fibrinogen concentration <2.0 g/L), type of extensive resection (hemispherectomy), allogeneic transfusion, fibrinogen concentrate administration, postoperative seizures and thromboembolic events, are expressed as N [percentages (%)] and compared between groups using the Pearson chi-square or continuity correction test as indicated. The relative risk (RR) and 95% confidence intervals (CI) between the two groups were calculated for data of allogeneic transfusion and postoperative seizures.

All statistical analyses were performed using SPSS software (version 20.0; SPSS Inc., Chicago, IL, United States), with a *p* value of <0.05 indicating statistical significance.

## Results

### Demographic and baseline characteristics

**FIGURE 2:**
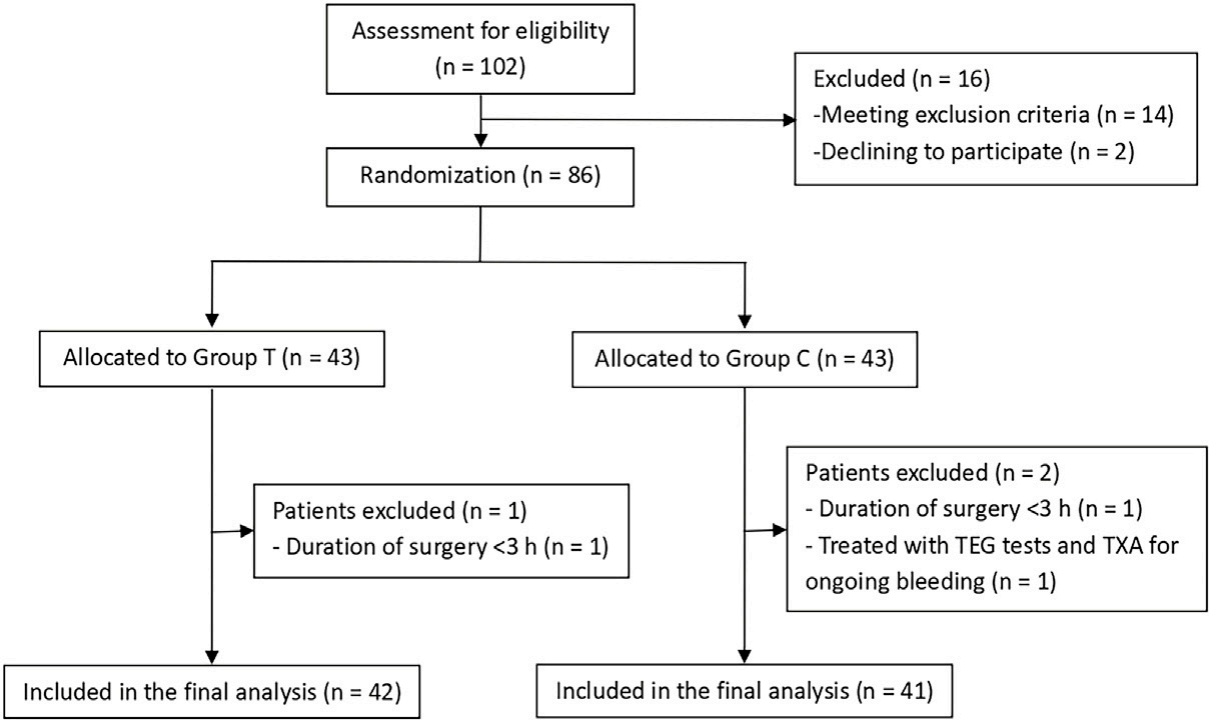
Flow chart of the study. TEG, thromboelastography; TXA, tranexamic acid.

A total of 102 patients were assessed for eligibility, and 16 patients were excluded prior to randomization. The 86 patients who satisfied the inclusion criteria were randomly and equally assigned to Groups T and C, with each group comprising 43 patients. One patient in each group was excluded from the analysis for having <3 h duration of surgery, whereas another patient in Group C was excluded due to TEG and TXA treatment for ongoing bleeding. Finally, 83 patients were included in the analysis (42 in Group T and 41 in Group C) (Figure 2).

Demographic and baseline characteristics of both groups are summarized in Table 1. Baseline parameters, such as age, height, weight, preoperative laboratory values, percentage of hemispherectomies, duration of surgery and anesthesia, were comparable between both groups. Mean preoperative hemoglobin values were 12.35 ± 0.91 and 12.79 ± 0.99 g/dl in Groups T and C, respectively, with no difference in preoperative anemia (hemoglobin concentration <12 g/dl) between both groups (*p* = 0.369) (Table 1). Moreover, 15 (35.7%) and 13 (31.7%) patients in Groups T and C had hypofibrinogenemia (plasma fibrinogen level <2.0 g/L) preoperatively, with no difference with regard to the percentage of preoperative hypofibrinogenemia between the two groups (*p* = 0.699) (Table 1).

**TABLE 1:** Demographic and baseline characteristics of the two groups.

Parameter	Group T (*n* = 42)	Group C (*n* = 41)	*p* value
Age^a^ (years)	6.08 ± 2.65	6.89 ± 3.00	0.194
Height^a^ (cm)	113.80 ± 16.01	121.21 ± 18.46	0.054
Weight^b^ (kg)	20.50 (18.75–26.00)	25.00 (18.75–29.50)	0.179
Preoperative anemia^c^ ^&^ [N (%)]	14 (33.3)	10 (24.4)	0.369
Preoperative laboratory values			
Platelets^a^ (10^9^/L)	273.17 ± 75.65	295.63 ± 84.26	0.205
PT^a^ (s)	13.24 ± 0.66	13.25 ± 0.76	0.970
APTT^b^ (s)	40.50 (36.55–44.03)	41.20 (36.45–44.40)	0.881
INR^a^	1.01 ± 0.07	1.01 ± 0.07	0.946
Preoperative hypofibrinogenemia^c^ ^&^ [N (%)]	15 (35.7)	13 (31.7)	0.699
Hemispherectomies^c^ ^#^ [N (%)]	3 (7.1)	2 (4.9)	1.000
Surgery duration^a^ (min)	282.60 ± 58.70	283.07 ± 73.75	0.974
Anesthesia duration^a^ (min)	394.48 ± 74.61	367.32 ± 79.01	0.111

a The values are given as mean ± SD, with groups compared by the independent-samples *t*-test.

b The values are given as median (25%–75% IQR), with groups compared by the Mann–Whitney *U*-test.

c The values are given as N (%), with groups compared by the ^&^Pearson chi-square test, or ^#^continuity correction test.

PT, prothrombin time; APTT, activated partial thromboplastin time; INR, international normalized ratio.

### Transfusion requirements

The requirements of allogeneic transfusion in Group T were significantly lower than that in Group C: intraoperative requirements (19.0% vs. 53.7%; RR 0.355, 95% CI 0.179–0.704; *p* = 0.001) and total perioperative requirements (31.0% vs. 56.1%; RR 0.552, 95% CI 0.326–0.934; *p* = 0.021) (Table 2). The risks of RBC transfusion were lower in Group T than in Group C; however, the differences were not statistically significant. Intraoperative and total risks of plasma exposure were significantly lower in Group T compared with Group C: intraoperative risks (7.1% vs. 51.2%; RR 0.139, 95% CI 0.045–0.432; *p* = 0.000) and total perioperative risks (19.0% vs. 53.7%; RR 0.355, 95% CI 0.179–0.704; *p* = 0.001) (Table 2). None of the patients in both groups required platelets transfusion. Further, 19 (45.2%) patients in Group T received fibrinogen concentrates guided by TEG data, whereas only 1 (2.4%) patient in Group C received fibrinogen concentrates because of a preoperative fibrinogen level of <1.5 g/L.

**TABLE 2:** Transfusion of blood products and fibrinogen concentrate.

Parameter	Group T (*n* = 42) [N (%)]	Group C (*n* = 41) [N (%)]	RR (95% CI)	*p* value
Risk of allogeneic transfusion^a^				
Intraoperative	8 (19.0)	22 (53.7)	**0.355 (0.179–0.704)**	**0.001**
Total^b^	13 (31.0)	23 (56.1)	**0.552 (0.326–0.934)**	**0.021**
Risk of RBC transfusion^a^				
Intraoperative	6 (14.3)	12 (29.3)	0.488 (0.202–1.177)	0.098
Total^b^	11 (26.2)	15 (36.6)	0.716 (0.374–1.369)	0.307
Risk of plasma transfusion^a^				
Intraoperative	3 (7.1)	21 (51.2)	**0.139 (0.045–0.432)**	**0.000**
Total^b^	8 (19.0)	22 (53.7)	**0.355 (0.179–0.704)**	**0.001**
Total platelets transfusion^b^	0 (0)	0 (0)	NA	NA
Fibrinogen concentrate infusion^a^	19 (45.2)	1 (2.4)	**18.548 (2.601–132.243)**	**0.000**

The values are given as N (%).

a Pearson chi-square test used to compare groups. Boldface type indicates statistical significance.

b Total includes intraoperative and postoperative transfusion.

RR, relative risk; CI, confidence interval; RBC, red blood cells; NA, not applicable.

### Surgical data and postoperative outcomes

Intra- and postoperative clinical outcomes and laboratory values are presented in Table 3. Accordingly, Group T intraoperatively received higher volumes of crystalloids than Group C. No significant differences were observed in terms of intraoperative colloid input, urine output, estimated blood loss, and postoperative values of hemoglobin and platelets in the groups. Postoperative hemoglobin levels decreased in both groups, with the reduction in hemoglobin from preoperative baseline to postoperative being comparable between both groups.

**TABLE 3:** Intraoperative and postoperative clinical outcomes and laboratory values in both groups.

Parameter	Group T (*n* = 42)	Group C (*n* = 41)	*p* value
Intraoperative fluids^a^			
Crystalloid (ml)	1490 (1325–1713)	1000 (700–1200)	**0.000**
Colloid (ml)	0 (0–200)	0 (0–200)	0.643
Urine output^a^ (ml)	850 (638–1093)	800 (500–1000)	0.247
Estimated blood loss^a^ (ml)	100 (100–155)	100 (100–150)	0.792
Hemoglobin level on POD 1^b^ (g/dl)	10.55 ± 1.31	10.58 ± 1.32	0.921
Delta hemoglobin^b^ (g/dl) (preoperative to POD 1)	–1.84 ± 1.39	–2.21 ± 1.67	0.290
Platelet count on POD 1^a^ (10^9^/L)	233.00 (185.00–263.00)	226.00 (186.50–286.50)	0.672
Postoperative thrombotic events^c^ [N (%)]	0 (0)	0 (0)	NA
Postoperative seizures^c^ [N (%)]	4 (9.5)	5 (12.2)	0.969
Hospital stay after surgery^a^ (days)	8.00 (7.00–11.25)	9.00 (7.50–11.00)	0.827

a The values are given as median (25%–75% IQR), with groups compared by the Mann–Whitney *U*-test. Boldface type indicates statistical significance.

b The values are given as mean ± SD, with groups compared by the independent-samples *t*-test.

c The values are given as N (%), with groups compared by the continuity correction test.

POD, postoperative day; NA, not applicable.

Both groups had comparable duration of hospital stay after surgery, with no thromboembolic events occurring in either group. Postoperative seizures of varying degrees occurred in four (9.5%) and five patients (12.2%) in Groups T and C, respectively, during their hospital stay, with no significant difference between the two groups (RR 0.781, 95% CI 0.225–2.705; *p* = 0.969) (Table 3).

### Coagulation profile

TEG tests were administered for all 42 patients in Group T. TEG values showed that 13 (31.0%) patients in Group T had accompanying preoperative fibrinogen dysfunction (K > 3 min or α < 53° at T1) and in 6 other patients, fibrinogen dysfunction occurred later during the surgery. After fibrinogen dysfunction had been corrected, three patients exhibited clotting factors deficiency in the TEG tests.

TEG data for the four time points (mentioned above) are reported in Table 4. Median R values were higher for T1 (8.20 min) than for T2 (7.85 min), T3 (7.45 min), and T4 (6.30 min), with the difference between T1 and T4 being significant (Table 4). Median K values were also higher for T1 (2.40 min) than for T2 (2.20 min), T3 (2.10 min), and T4 (1.80 min), with differences reaching significance for all comparisons (Table 4). The median α angle values for T2 (63.50°), T3 (62.25°), and T4 (65.10°) were significantly higher than those for T1 (60.60°) (*p* < 0.05 for all), with similar findings observed for MA values (Table 4). Overall, changes in TEG values showed that the hemostatic system was enhanced over time during the surgery. LY30 values were significantly lower at T4 than at T1; however, all LY30 values were within normal limits, indicating that none of the patients in Group T developed hyperfibrinolysis. No intraoperative standard coagulation tests were performed in Group C.

**TABLE 4:** TEG data in Group T.

TEG variable	T1	T2	T3	T4	*p* value	*p* value	*p* value
					T2 vs. T1	T3 vs. T1	T4 vs. T1
R (min)	8.20 (7.10–9.35)	7.85 (7.20–9.23)	7.45 (6.65–9.23)	6.30 (5.20–8.90)	0.757	0.576	**0.006**
K (min)	2.40 (1.95–3.00)	2.20 (1.80–2.53)	2.10 (1.80–2.73)	1.80 (1.60–2.50)	**0.017**	**0.004**	**0.010**
α angle (degrees)	60.60 (53.15–66.25)	63.50 (58.70–66.33)	62.25 (59.60–66.33)	65.10 (57.10–69.70)	**0.024**	**0.028**	**0.005**
MA (mm)	56.80 (52.25–60.60)	57.95 (55.38–61.60)	58.60 (53.58–61.60)	59.90 (56.40–63.00)	**0.030**	**0.025**	**0.009**
LY30 (%)	0.50 (0.10–1.50)	0.55 (0.00–1.00)	0.25 (0.00–0.80)	0.20 (0.00–0.80)	0.334	0.189	**0.021**

The values are given as median (25%–75% IQR). The *p* values are obtained using the Wilcoxon signed-rank test. Boldface type indicates statistical significance.

TEG, thromboelastography; R, reaction time to clot formation; K, coagulation time; α angle, angle of divergence; MA, maximum amplitude of clot strength; LY30, percentage lysis 30 min post-MA; T1, prior to incision; T2, cutting of the dura mater; T3, suturing of the dura mater; T4, after scalp closure.

## Discussion

The present study demonstrated that allogeneic transfusion requirements during pediatric resective epilepsy surgery reduced by 34.7% (RR of 0.355) with the use of an intraoperative multimodal coagulation therapy. The total risk of perioperative allogeneic blood exposure in Group T was also reduced by 25.1% (RR of 0.552), and this did not translate to lower postoperative hemoglobin concentrations. Moreover, no changes in postoperative outcomes of seizures or thromboembolic events were observed between the two groups. These findings suggest that the prophylactic administration of TXA combined with TEG-guided goal-directed hemostatic algorithm is an effective multimodal coagulation strategy for reducing allogeneic transfusion requirements during pediatric resective epilepsy surgery.

TEG is a viscoelastic test that can provide timely clotting and fibrinolysis information in the operation theater and guide direct supplementation of indicated blood components for appropriate coagulopathy (Whiting et al., 2015). The interpretation of TEG-5000 parameters from the User’s Manual is summarized in Table 5. TEG-guided component replacement has been described in a variety of previous reports on bleeding management (Walsh et al., 2011; Johansson et al., 2013; Christiaans et al., 2014; Kane et al., 2016; Sujka et al., 2018). The time to clot formation (R-time) was used as a guide for clotting factors, the speed of clot formation (K-time, or α angle) as an indication for fibrinogen level. It was reported that ∼80% of the clot strength was due to platelet contribution, and the remainder was the contribution of fibrin network (Wegner and Popovsky, 2010). Therefore, in the presence of normal fibrinogen level, overall clot strength (MA) was used to assess platelet function (Tanaka et al., 2014).

**TABLE 5:** Interpretation of TEG-5000 parameters.

Main parameter	Definition	Coagulation correlation	References ranges
R	The time from initiation to initial fibrin formation, arbitrarily defined as the trace amplitude of 2 mm	Clotting factors	5–10 min
K	The time taken for the amplitude to increase from 2 to 20 mm	Fibrinogen	1–3 min
α angle	The angle formed between the midline and the tangent to the main body of the trace	Fibrinogen	53°–72°
MA	The amplitude at the widest point of the trace	Platelet (∼80%) Fibrinogen (∼20%)	50–70 mm
LY30	The percentage reduction in amplitude 30 min after MA is reached	Fibrinolysis	0%–8%

Reference ranges according to the manufacturer of TEG-5000, for kaolin-activated citrated and recalcified blood samples.

R, reaction time to clot formation; K, coagulation time; α angle, angle of divergence; MA, maximum amplitude of clot strength; LY30, percentage lysis 30 min post-MA.

In the current study, TEG data revealed that 13 (31.0%) patients in Group T had accompanying preoperative fibrinogen dysfunction and 6 other patients experienced fibrinogen dysfunction later during surgery. After fibrinogen dysfunction had been corrected, only three patients exhibited clotting factors deficiency. Our results demonstrated that fibrinogen dysfunction was the main coagulopathy in pediatric resective epilepsy surgery. This could have been due to preoperative AEDs administration, which can impair hemostatic system, including a decrease in fibrinogen (Gerstner et al., 2006). Consistent with the mentioned finding, our laboratory results showed that approximately 1/3 of all patients showed complications of preoperative hypofibrinogenemia. Another reason for fibrinogen dysfunction may be craniotomy and intraoperative bleeding, considering Levy et al.’s (2012) finding that fibrinogen was the first coagulation factor that achieved a critical low value during massive blood loss. Evidence has shown that fibrinogen deficiency could further aggravate perioperative bleeding after craniotomy (Adelmann et al., 2014). Therefore, fibrinogen supplementation is vital for reversing and maintaining hemostatic function for pediatric epilepsy patients. Guided by TEG values, patients accompanied with fibrinogen dysfunction in Group T received directed fibrinogen concentration replacement. Thereafter, the hemostatic system was obviously optimized, as evidenced by a reduction in the K time and increase in the α angle and MA value.

The currently recommended threshold for perioperative fibrinogen replacement is 1.5–2.0 g/L (Kozek-Langenecker et al., 2017). Regarding the risk of increased intraoperative bleeding, one patient in Group C received fibrinogen concentrate for preoperative plasma fibrinogen level of <1.5 g/L, whereas other patients with preoperative hypofibrinogenemia (plasma fibrinogen concentrations ranging 1.5–2.0 g/L) received plasma transfusion. Since laboratory coagulation values could only be obtained in a long period of >2 h at our center, no intraoperative standard coagulation tests were performed in Group C. Intraoperative plasma transfusion in Group C was performed at the discretion of the attending anesthesiologists, mainly depending on signs of recurrent bleeding from the wound margins. Overall, 21 (51.2%) patients in Group C were transfused plasma traditionally to correct abnormal coagulation function and control microvascular bleeding. Generally, plasma is primarily recommended to correct coagulation factor deficiencies (Kozek-Langenecker et al., 2017). Studies have shown that plasma transfusion alone is inadequate to correct hypofibrinogenemia (Kozek-Langenecker et al., 2017). Bolliger et al. (2010) found that a minimum FFP transfusion volume of 20–30 ml/kg should be administered before expecting a significant increase in plasma fibrinogen concentration, whereas the frequently recommended FFP dosage ranges 10–15 ml/kg (Goobie and Haas, 2014). Fibrinogen concentrate, associated with decreased immunogenic and infectious complications and rapid availability (Levy et al., 2012), has recently been recommended to correct hypofibrinogenemia among pediatric surgery cases (Kozek-Langenecker et al., 2017). In the present study, after directed fibrinogen concentration replacement, only three (7.1%) patients in Group T required plasma transfusion because of clotting factors deficiency. Therefore, with the implementation of TEG-guided hemostatic algorithm, the risk of intraoperative plasma exposure was greatly reduced (by 44.1%, RR of 0.139). Our results are in accordance with the findings of Ak et al. (2009) and Schaden et al. (2012), where Ak et al. (2009) observed that a TEG-guided transfusion algorithm promoted a significant lower FFP exposure compared to physician-directed transfusion in patients undergoing elective coronary artery bypass grafting, and Schaden et al. (2012) reported a significant reduction in allogeneic transfusion after a viscoelastic testing-based coagulation strategy for bleeding burn patients. In addition, in the present study, as plasma transfusion was significantly reduced, more crystalloid was needed to replace and maintain intravascular volume in Group T (Table 3).

As an essential component of multimodal coagulation therapy, TXA has been the most widely recommended pharmacological agent for suppressing fibrinolysis and preventing perioperative bleeding in recent pediatric critical bleeding guidelines (Kozek-Langenecker et al., 2017; Goobie et al., 2019). However, only a few clinical trials are available on TXA administration for pediatric epilepsy surgery, which might be owing to the concern of side effects of TXA, particularly seizures. Evidence has suggested that TXA-associated seizures are dose-related, given that high TXA dosing schemes, such as 100 mg/kg or >2 g/d have been reportedly associated with seizures in high-risk patients who underwent cardiac surgery or those with pre-existing renal impairment (Murkin et al., 2010; Lin and Xiaoyi, 2016; Murao et al., 2021). Moreover, studies using low dosage regimens did not report increased risk of seizures in pediatric patients (Goobie et al., 2017; Faraoni et al., 2019; Murao et al., 2021). In our trial, we cautiously selected a lower dosage scheme (10 mg/kg loading dose followed by 5 mg/kg/h maintenance infusion) based on pharmacokinetic modeling in pediatric patients, which had been shown to produce stable and therapeutic TXA plasma concentrations (Goobie et al., 2013). We found no significant difference in the incidence of postoperative seizures between the two groups, which agree with the findings of Goobie et al. (2017) during pediatric craniofacial surgery. None of the patients in Group T developed a clinical thromboembolic event, which is consistent with the finding that appropriate TXA administration was not associated with increased risk of thrombotic events (Shakur et al., 2010; Shakur et al., 2017; Myles et al., 2017; Goobie and Faraoni, 2019; Roberts et al., 2019; Murao et al., 2021). These results indicate that the TXA dosage regimen we adopted could be well tolerated by pediatric epilepsy patients.

LY30 values, the gold standard in detecting hyperfibrinolysis (Haas et al., 2014), were all within normal ranges (<8%), demonstrating that no hyperfibrinolysis occurred in Group T. In addition, the LY30 values at T4 were significantly lower than that at T1, indicating that fibrinolysis was further inhibited by TXA administration. Our results confirmed that prophylactic TXA administration could be an effective coagulation therapy for pediatric epilepsy surgery.

Data regarding RBC transfusion in pediatric resective epilepsy surgery are scarce. Most studies were retrospective and reported risks of RBC exposure that varied from 30% to 51% (Manohar et al., 2011; Thudium et al., 2014; Vadera et al., 2015). In our study, the total perioperative RBC transfusion requirements were approximately 30%, which was comparable to the findings of Manohar et al. (2011) and Vadera et al. (2015) but significantly lower than the 51% reported by Thudium et al. (2014). An important reason underlying this might be the different settings of RBC transfusion trigger; this value was 8 g/dl in our trial and 9.5 g/dl in the study by Thudium et al. (2014). The restrictive hemoglobin threshold of 8 g/dl has been shown to be indicated and safe in pediatric craniotomies (Goobie and Haas, 2014). In our study, the requirements for RBC transfusion were lower in Group T than in Group C, which may confirm the efficacy of our multimodal coagulation therapy in reducing RBC exposure. Given that our sample size was calculated based on transfusion incidence of all allogeneic products, including RBC and plasma, more patients may be needed to achieve a statistical difference.

Pediatric resective epilepsy surgery has been associated with significant blood loss (Thudium et al., 2014). In our study, the median estimated blood loss (EBL) was 100 ml for both groups, which was lower than the value (150 ml) reported by Thudium et al. (2014). This may be because fewer complex surgeries (only three hemispherectomies in Group T vs. two in Group C) had been conducted in recent years at our center, given that hemispherectomies had a significantly higher EBL than single lobectomies (Manohar et al., 2011). In addition, advancements in microsurgical techniques and the extensive experience of our surgical team might also contribute to the decreased blood loss. No significant difference was observed in the EBL between the groups, perhaps due to two factors: 1) blood loss itself is difficult to evaluate accurately and 2) surgical and anesthesia techniques have been constantly refined given that the current study lasted for over 3 years, and blood loss in these surgical settings at our center has been lower than before; this suggests that a larger sample size might be needed to detect differences in blood loss.

The current study has several limitations. First, given that this coagulation management strategy is a bundled treatment protocol, the reduction in allogeneic transfusion in our study cannot be attributed to one specific treatment factor, and further studies separately evaluating the efficacy of TXA administration or TEG-guided hemostatic algorithm for pediatric resective epilepsy surgery are warranted. Second, no TEG analyses were performed in Group C; thus, we could not compare the difference in coagulation function between groups due to the lack of matching TEG values in Group C. Therefore, the conclusion that our multimodal coagulation therapy can activate the hemostatic system of pediatric epilepsy surgical patients should be interpreted with caution. Moreover, intracranial surgery itself could be combined with enhanced hemostatic system given that the injured brain could release tissue thromboplastin and activate the coagulation cascade (van der Sande et al., 1983). Third, although the recommended triggers of TEG parameters for coagulation therapy have increased over the last years, a universally accepted threshold for initiating clotting factor infusion, as well as the dose required to reach the targeted level, are still missing, especially for pediatric craniotomies. This needs to be verified by future clinical trials. Finally, while no thromboembolic events or increased incidence of postoperative seizures were observed in our study, it was not powered to make conclusions on safety of the multimodal coagulation therapy. Future larger multicenter trials in pediatric epilepsy surgery would be required to confirm the safety profile.

In conclusion, this study demonstrated that fibrinogen dysfunction was the main coagulopathy during pediatric resective epilepsy surgery. As a multimodal coagulation therapy for pediatric resective epilepsy surgery, the prophylactic use of TXA combined with TEG-guided hemostatic algorithm can optimize hemostatic function and promote lower risk of allogeneic exposure compared to conventional coagulation management strategies.

## Data Availability

The original contributions presented in the study are included in the article/supplementary material, further inquiries can be directed to the corresponding author.
